# Another common genetic ataxia in South Korea: Spinocerebellar ataxia 36

**DOI:** 10.1038/s41431-024-01783-9

**Published:** 2025-02-24

**Authors:** Jong Hyeon Ahn, Seungbok Lee, Jangsup Moon, Yoojung Han, Hyeshik Chang, Jinyoung Youn, Jin Whan Cho, Ja-Hyun Jang

**Affiliations:** 1 Department of Neurology, Samsung Medical Center, Sungkyunkwan University School of Medicine, Seoul, South Korea; 2 Neuroscience Center, Samsung Medical Center, Seoul, South Korea; 3 Department of Genomic Medicine, Seoul National University Hospital, Seoul, South Korea; 4 Department of Pediatrics, Seoul National University College of Medicine, Seoul National University Children’s Hospital, Seoul, South Korea; 5 Department of Neurology, Seoul National University Hospital, Seoul, South Korea; 6 Center for RNA Research, Institute for Basic Science (IBS), Seoul, South Korea; 7 Interdisciplinary Program in Bioinformatics, Seoul National University, Seoul, South Korea; 8 School of Biological Sciences, Seoul National University, Seoul, South Korea; 9 Department of Laboratory Medicine and Genetics, Samsung Medical Center, Sungkyunkwan University School of Medicine, Seoul, South Korea

**Keywords:** Movement disorders, Genetics research

## Abstract

Spinocerebellar ataxias (SCAs) represent a diverse group of neurodegenerative disorders characterized by progressive cerebellar ataxia. In South Korea, diagnostic laboratories typically focus on common SCA subtypes, leaving the prevalence of rare SCAs uncertain. This study aimed to explore the frequency of rarer forms of SCA, including SCA10, 12, 31, and 36 utilizing molecular techniques including long-read sequencing (LRS). Patients from ataxia cohorts who remained undiagnosed after testing for common genetic ataxias (SCA1, 2, 3, 6, 7, 8 17, and dentatorubral-pallidoluysian atrophy) were analyzed, along with unselected ataxia patients referred for screening of common SCAs. Expanded alleles for SCA10, 12, 31, and 36 were investigated through allele-length PCR, repeat-primed PCR, and LRS. Among 78 patients from 67 families with undiagnosed cerebellar ataxia, SCA36 was identified in 8 families (11.9%), while SCA10, 12, or 31 were not found. In unselected ataxia, SCA36 was present in 1.0% (1/99). Korean SCA36 patients exhibited clinical characteristics similar to global reports, with a higher incidence of hyperreflexia. The haplotype of expanded alleles identified in LRS was consistent among SCA36 patients. The findings indicate that SCA36 accounts for 11.9% of diagnoses after excluding common SCAs and 1.0% in unselected ataxia patients. The study underscores the prevalence of SCA36 in South Korea and emphasizes the potential of LRS as a diagnostic tool for this condition. Integrating LRS into diagnostic protocol could enhance diagnostic efficacy, particularly in populations with a high prevalence of SCA36 like South Korea. Further research is necessary to standardize LRS for routine clinical application.

## Introduction

Spinocerebellar ataxias (SCAs) are a group of heterogeneous neurodegenerative diseases characterized by progressive cerebellar ataxia with an autosomal dominant inheritance pattern. Until now fifty types of SCAs have been identified, with twelve attributed to repeat expansions [1, 2]. While SCA3 stands as the commonly encountered subtype on a global scale [3], the prevalence of specific genetic ataxia can vary considerably depending on ethnic and geographic factors. Previous studies conducted in South Korea have identified either SCA2 or SCA3 as the most common subtypes, depending on the literature [4–8].

Despite previous knowledge regarding the prevalence of SCAs, this understanding may be incomplete due to challenges in comprehensive testing, attributed to cost and labor constraints. Additionally, the diagnosis of certain SCAs poses difficulties due to the presence of long repeat expansions. Repeat-primed PCR (RP-PCR) has been used as a valuable diagnostic tool for SCAs, especially when conventional fluorescent amplicon-length PCR (AL-PCR), which amplifies the target region using flanking primers and separates and sizes them by capillary electrophoresis, fails to amplify expanded alleles containing large repeat numbers. However, for certain specific SCAs such as SCA10 and SCA36, a saw-tooth pattern in RP-PCR cannot be interpreted as a full mutation due to the broad range of alleles of uncertain significance between normal and full mutations. Discriminating between full mutations and alleles of uncertain significance might be possible by measuring the repeat size. In such cases, diagnosis has traditionally relied on the labor- and resource-intensive Southern blotting technique, presenting practical challenges in clinical settings [1].

The advent of next-generation sequencing (NGS) technologies has enhanced diagnostic capabilities [9], and with progress in bioinformatics, detecting the most common disease-causing repeat expansions has also become reliable even with short-read sequencing data [10, 11]. However, accurately sizing repeats that exceed the read length remains a challenge. Long-read sequencers (LRS) such as single-molecule real-time (SMRT) sequencing, or Oxford Nanopore Technologies (ONT) offer promise as they enable real-time single DNA molecule sequencing with their long read-length [12]. This technology has been utilized in the diagnosis of various genetic diseases, including repeat expansion diseases, and has benefits compared to the NGS and Southern blot at once, without radiation, technique [12].

In the majority of routine diagnostic laboratories in South Korea, the test is typically limited to SCA1, 2, 3, 6, 7, 8 and 17 as these are commonly associated with ataxia among Korean patients. We already investigated the prevalence of genetic ataxia in ataxic patients using whole exome sequencing, but the positive rate was not high enough when sequence variations were analyzed [13]. Repeat expansions can be detected through exome sequencing [14, 15], but it remains challenging when the repeat size in the patient is large or when the expansion is located in a non-coding region. Therefore, the prevalence of less common SCAs is still largely unknown. In the present study, we aimed to investigate the prevalence of SCA10, 12, 31, and 36 in patients with undiagnosed cerebellar ataxia in South Korea. Additionally, we validated the prevalence using unselected ataxia patients. To achieve this, we employed various molecular techniques, including LRS, specifically tailored to the repeat structure and/or size of the implicated genes.

## Methods

### Participants

Participants in cohort 1 consisted of undiagnosed ataxia patients from the ataxia cohorts of the Neurology Clinics at both Samsung Medical Center and Seoul National University Hospital from March 2020 to October 2022. These individuals were suspected of having a genetic cause for the ataxia but remained undiagnosed following extensive genetic testing. Acquired causes of ataxia were ruled out in all participants after comprehensive clinical evaluation for acquired causes of ataxia before consideration of genetic causes. For enrollment in this study, patients were required to have negative testing for the most common genetic ataxias in Korea, specifically SCA1, SCA2, SCA3, SCA6, SCA7, SCA8 and SCA17 and dentatorubral-pallidoluysian atrophy (DRPLA). The study did not exclude patients based on age of onset, mode of inheritance, or family history to avoid bias against phenotypic variability and inaccurate assessment of familial association in cases where clinical assessment of relatives was not possible. For this study, age at onset was determined based on patient or caregiver statements regarding the onset of symptoms. Clinical characteristics including severity of ataxia were assessed. The severity of ataxia was assessed using the validated Scale for the Assessment and Rating of Ataxia (SARA), which scores on a 40-point scale with 0 indicating normal function [16].

To ascertain the prevalence of SCA among undiagnosed ataxia patients (cohort 1), an additional cohort of patients with ataxia (cohort 2) was tested, consisting of individuals referred to the Samsung Medical Center for genetic testing for ataxia diagnosis from January 2011 to February 2022. Residual DNA was anonymized after the completion of ordered tests and included in the study.

The Institutional Review Board of Samsung Medical Center and Seoul National University Hospital approved this study. All enrolled subjects in cohort 1 provided written informed consent. The requirement for informed consent was waive for cohort 2 due to a retrospective nature.

### Screening methods

For the analysis of 4 loci (SCA10, 12, 31, and 36), different strategies were used depending on the repeat ranges of full penetrance alleles as well as repeat structures (Table S1). Types of the testing method and primers of each locus were based on previous literature, as detailed in Table S2 [17–21]. PCR products of AL-PCR and RP-PCR were separated on an ABI 3730xl DNA analyzer (Applied Biosystems–Thermo Fisher Scientific, Foster City, CA) and analyzed through Microsatellite Analysis software (Applied Biosystems–Thermo Fisher Scientific, Foster City, CA). Briefly, in the case of SCA12, where the repeat number of the full penetrance allele is not large, if homoallelism is detected in AL-PCR, RP-PCR is performed to confirm whether the unamplified expanded allele is present or not by looking for the saw-tooth pattern. For SCA10 and SCA36, the process is identical to SCA12 except repeat number estimations of the expanded allele are required for the distinction between pathogenic and uncertain significance. In the present study, LRS was utilized to confirm repeat size when a saw-tooth pattern was detected in RP-PCR. Pathogenic SCA31 alleles exhibit insertions ranging from 2.6 to 3.7 kb in length, featuring (TGGAA)n stretch at their 5′-end. To screen for these insertions, PCR amplification followed by HaeIII enzyme digestion was employed, wherein alleles lacking an insertion would yield a short fragment. In cases where an insertion was detected, RP-PCR was supposed to be conducted to confirm the presence of TGGAA repeats, as there may be rare individuals carrying a long insertion lacking this repeat.

### Long-read sequencing (LRS)

LRS using the ONT platform was performed to determine the exact repeat number of expanded alleles for SCA36. In the case of these two subtypes, solely detecting an expanded allele in RP-PCR isn’t sufficient to distinguish between a pathogenic allele and one with uncertain significance.

The target region of *NOP56* was enriched with the CRISPR-Cas9 system with ligation kit R9.4 version (ONT, SQK-LSK109,110) and according to cas9 targeted sequencing protocol (ONT, version ENR_9084_v109_revD_04Dec2018). CRISPR RNA (crRNA) targeting *NOP56* was designed with CHOPCHOP covering 4.2 kb around the repeat region (coordinates: chr20: 2650703-2654951, GRCh38) (Table S2). Prepared libraries were sequenced on MinION flowcells (Oxford Nanopore technology) until a plateau was reached. Base calling was performed through Dorado (‘dna_r9.4.1_e8_sup@v3.6’ model), with adjustments for long repeats (‘–batchsize 128 –chunksize 80000’). Sequence reads were mapped to the GRCh38.p13 human reference genome using Dorado (options: ‘-k 13 -w 20 -s 40’). Repeat counts are derived from the alignment files using RepeatHMM (options: ‘–MinSup 0 –SplitAndReAlign 2 –SeqTech Nanopore –outlog DEBUG’) [22]. Instead of RepeatHMM’s standard output, verbose debugging output is used to extract the unprocessed intermediate information. The repeat counts measured in the read level are log-transformed and fitted to a two-Gaussian mixture model, selecting the median of each cluster as the allele’s repeat length.

### Statistical analysis

The demographic and clinical characteristics of the participants were expressed using means and standard deviations (SD). To discern differences between patients in our study and SCA36 patients worldwide, we employed the chi-square test, Student’s *t*-test, or Fisher’s exact test as appropriate. A *p*-value of < 0.05 was considered statistically significant. Statistical analysis was performed with the IBM SPSS (version 28.0; IBM Inc., USA) software for Windows.

## Results

### Screening of SCA10, 12, 31, and 36 with undiagnosed cerebellar ataxia (cohort 1)

**Fig. 1:**
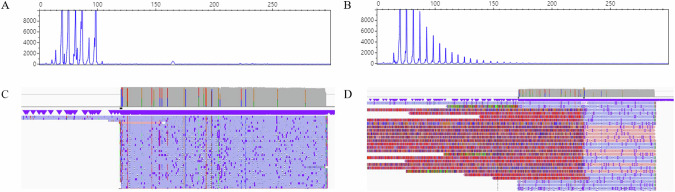
Illustration of examples of test results for *NOP56* hexanucleotide repeat expansions for the diagnosis of SCA36. **A** Negative result in repeat-primed PCR (RP-PCR) screenings. **B** Typical saw-tooth pattern in RP-PCR indicating the presence of the expanded allele. **C** Negative result in long-read sequencing (LRS) using Oxford Nanopore Technologies. **D** LRS result showing a large insertion in intron of *NOP56*.

We recruited a cohort comprising 78 cerebellar ataxia patients from 67 families (cohort 1) who had not received a diagnosis through testing for the most common genetic ataxia via repeat expansions. These individuals underwent screening for SCA10, 12, 31, and 36. No insertion were observed in PCR amplification followed by HaeIII enzyme digestion for SCA31. Regarding SCA10, 12, and 36, the heterozygosity percentages by AL-PCR were 77% (60/78), 81% (63/78), and 50% (39/78), respectively and their repeat number distributions at each locus are depicted in Fig. S1. For cases displaying homoallelism in SCA10 and SCA12, RP-PCR was performed, but no instances of a saw-tooth pattern were observed. For SCA36, RP-PCR revealed a saw-tooth pattern in RP-PCR in ten patients from eight families, indicating the presence of expanded alleles beyond the normal repeat range shown in Fig. 1. This accounted for 11.9% of the families with undiagnosed cerebellar ataxia, whose characteristics are outlined in Table 1.

**Table 1:** Demographics and clinical characteristics of SCA36 patients.

	A-1	A-2	B	C-1	C-2	E	F	G	H	I
Age of examination, y	66	69	63	62	71	66	58	51	65	55
Age of onset, y	49	68	53	48	53	55	42	48	55	51
Sex	F	F	F	M	M	F	M	F	F	F
Repeat numbers	na	na	na	793	na	908	1403	537	890	928
Family history	+	+	+	+	+	+	+	+	+	+
Truncal ataxia	+	+	+	+	+	+	+	+	+	+
Limbs ataxia	+	+	+	+	+	+	+	+	+	+
Saccadic pursuit	na	na	na	+	+	+	+	+	-	+
Ataxic dysarthria	+	+	+	+	+	+	+	+	+	+
Tongue atrophy and fasciculation	na	na	na	-	na	+	-	+	-	+
Muscle atrophy and fasciculation	-	-	-	-	na	-	-	+	-	-
Hyperreflexia	+++	+++	+++	+++	+++	+++	+++	+++	+++	+++
Hearing impairment	+	+	+	+	+	+	+	+	-	-
Cognitive impairment	-	-	-	-	+	-	-	-	+	-
SARA score	9.5	na	na	na	na	6	22	8.5	6	24
Cerebellar atrophy	+	+	+	+	+	+	+	+	+	+

The data included only SCA36 patients from cohort 1, as clinical information for cohort 2 was not available.

*+* positive, *−* negative, *na* not available, *SARA* Scale for Assessment and Rating of Ataxia.

### Prevalence of SCA36 in unselected ataxia patients (cohort 2)

Due to the unexpectedly high positive rate in cohort 1, we conducted further study investigate the prevalence of SCA36 in independent ataxia cohort (cohort 2). Between January 2011 and February 2022, 99 samples were referred to the Department of Laboratory Medicine and Genetics in Samsung Medical Center for the genetic evaluation of common SCAs including SCA1, SCA2, SCA3, SCA6, SCA7, SCA8, and SCA17. Tests were performed based on physicians’ orders. Table S5 summarizes the tested items and their results. While 84 samples were tested for all seven loci, 11 samples were tested only for SCA17, three samples only for SCA8, and one sample only for SCA7. In the case where only SCA8 or SCA17 was requested, it is highly likely that the remaining SCA subtypes were found to be negative in the previous test, so only the remaining subtypes that were not tested were requested. Consequently, 12 samples (12.1%) were diagnosed with common SCAs; seven with SCA2, three with SCA8, one with SCA1, and one with SCA7, respectively. A total of 87 samples undiagnosed with common SCAs underwent AL-PCR and RP-PCR for SCA36. Among these samples, we identified a saw-tooth pattern in RP-PCR in one sample (ATA-109), accounting for 1.0% (1/99) of unselected ataxia patients.

### Confirmation of SCA36 using LRS

**Fig. 2:**
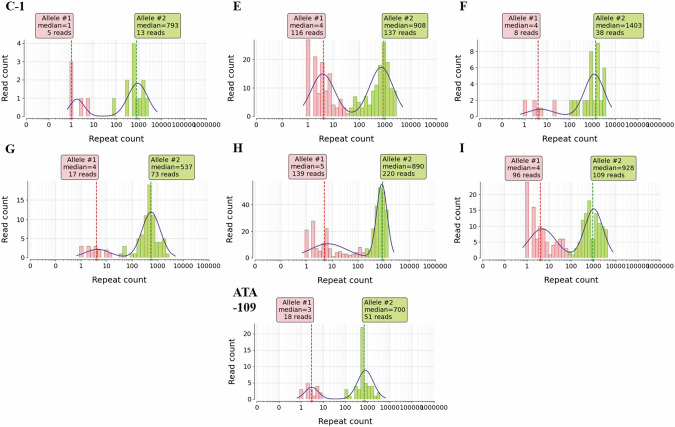
Estimated repeat numbers measured by long-read sequencing in SCA36 patients. The initials at the top of each figure point to the subject identification. The repeat counts measured in the read level are log-transformed and fitted to a two-Gaussian mixture model, selecting the median of each cluster as the allele’s repeat length. C-1, E, F, G, H, and I are samples belonging to the ataxia cohort, and ATA-109 is one of the samples that were referred for genetic evaluation of ataxia. These are the cases in which LRS was performed because expansion was suspected in repeat-primed PCR.

Among the 11 patients (10 from cohort 1 and one from cohort 2) exhibiting a saw-tooth pattern in SCA36 RP-PCR, seven (six from cohort 1 and one from cohort 2) underwent LRS. The depth of coverage within the target region varied from 46-845X, representing approximately 1 ~ 2% of the total obtained reads (Table S3). The estimated repeat number ranged from 537-1403 (Fig. 2) and their repeat structures were depicted in Fig. S2. Despite strong indications of abnormal *NOP56* repeat expansions based on their RP-PCR results, LRS could not be applied to the remaining patients due to DNA availability constraints. The expanded alleles showed the same haplotype within 4.2 kb region which was targeted by crRNAs (Table S4).

### Clinical characteristics of Korean SCA36 patients

In this study, we conducted a comparison of clinical characteristics between SCA36 patients in our cohort and those reported in a previous review by Wang et al., which included 161 patients from various international studies (Table 2) [23]. There were no differences in the age of examination, age of onset, and sex between the Korean SCA36 patients and the worldwide SCA36 patients. In terms of clinical characteristics, no substantial differences were found between the Korean and worldwide groups, except that the Korean patients had more frequent hyperreflexia than worldwide patients (100% vs. 56.3%, *p* = 0.006).

**Table 2:** Comparison between Korean SCA36 patients from ataxia cohorts and worldwide SCA36 patients.

	Korean (Present study, *n* = 10)	Worldwide (*n* = 161)	*p*-value
Age of examination, mean (SD)	62.6 (6.3)	62.8 (10.4)	0.955
Age of onset, mean (SD)	52.2 (6.8)	51.2 (7.3)	0.684
Sex, male (%)	30.0% (3/10)	48.1% (74/154)	0.338
Family history	100.0% (10/10)	na	–
Truncal ataxia	100.0% (10/10)	99.3% (144/145)	>0.999
Appendicular ataxia	100.0% (10/10)	96.1% (144/145)	>0.999
Saccadic pursuit	85.7% (6/7)	80.7% (46/57)	>0.999
Ataxic dysarthria	100.0% (10/10)	91.5% (130/142)	>0.999
Tongue atrophy and fasciculation	50.0% (3/6)	56.3% (71/126)	>0.999
Muscle atrophy and fasciculation	11.1% (1/9)	32.9% (28/85)	0.266
Hyperreflexia	100.0% (10/10)	56.3% (71/126)	0.006
Hearing impairment	80.0% (8/10)	56.3% (67/104)	0.490
Cognitive impairment	20.0% (2/10)	25.6% (11/43)	>0.999
SARA score	17.7 (5.5)	17.1 (*n* = 58)	0.851
Cerebellar atrophy	100.0% (10/10)	na	–

The data included only SCA36 patients from cohort 1, as clinical information for cohort 2 was not available. Significance was set at *p*-value < 0.05. *SARA* Scale for Assessment and Rating of Ataxia.

## Discussion

The present study investigated the prevalence of SCA10, 12, 31, and 36 using comprehensive methodologies, including RP-PCR and LRS based on the repeat ranges of full penetrance alleles as well as repeat structures. As a result, expanded alleles of SCA36 were identified in 11.9% of undiagnosed cerebellar ataxia families (cohort 1) and 1.0% of unselected ataxia patients (cohort 2). These findings underscore the potential underdiagnosis of SCA36, especially in Korean populations, due to the lack of a convenient diagnostic method. In addition, our study unveiled distinct clinical features in the Korean SCA36 patients, distinguishing them from their global counterparts. Notably, Korean patients exhibited a heightened frequency of hyperreflexia.

SCA36 has been discovered across diverse populations, with its prevalence in genetic ataxia cohorts ranging from 0.3% to 6.3% [20, 21, 24–28]. Interestingly, some studies have failed to detect SCA36 in certain European countries, including Germany, Greece, and Portugal [29]. However, the prevalence of SCA36 has been found to be particularly high in the Galicia region of Spain (6.3%) [27], and also high in the Japan (1.5% of all Japanese SCAs) [20] and China (1.6% of autosomal dominant SCAs) [26]. A recent study found that SCA36 accounted for 0.5% of cases of hereditary ataxia in the British population [28]. Our current investigation revealed that the presence of SCA36 in 11.9% of cohort 1 and 1.0% of cohort 2. The frequency observed in cohort 1 significantly surpasses that reported in other populations. This higher frequency may be attributable to the exclusion of other common SCAs, and some of the patients were ruled out for other genetic conditions through exome sequencing. Moreover, the inclusion of patients with a family history of ataxia may have contributed to the observed prevalence. Although not all patients’ family histories were available, at least all patients diagnosed had a family history. The prevalence observed in cohort 2, which includes unselected ataxia patients, may align more closely with findings from previous studies in Japan and China. However, further studies utilizing a larger number of patients would be necessary, as cohort 2 may not sufficiently represent the Korean ataxia patients, especially considering that SCA3, one of the most prevalent subtypes, was not observed in cohort 2. Furthermore, given the reported founder effect in specific regions such as the area around the Asida River of Japan and the Galicia region of Spain for SCA36 [20, 21, 27], there is a possibility of genetic relatedness among our patients. In this study, all patients exhibited the same haplotype within a 4.2 kb region, where target enrichment was performed, including several common single nucleotide polymorphisms that have been previously described in Japanese, French, and Han Chinese [20, 24]. However, further research covering a broader genomic region and involving more patients is needed to explore the potential founder mutation and the genetic association among other populations. In terms of clinical features, Korean patients with SCA36 demonstrated a higher prevalence of hyperreflexia, and SCA36 patients in China and Western Japan also reported a higher prevalence of hyperreflexia from 79 to 100%, which supports the forementioned genetic relatedness.

This study could not identify any patients with SCA10, 12, or 31, consistent with the previous literature findings. SCA10 is recognized as the most common SCA in Peru, the second most common subtype in Mexico, and prevalent in certain regions of Southern Brazil [30]. However, its occurrence in other regions is known to be rare, although isolated case reports have been documented in Japan and China [31–33]. Similarly, SCA12 is predominantly found in a single ethnic group from northern India [34], while a very limited number of cases have been reported in China [35]. While SCA31 is known as a prevalent ataxia in Japan [18, 36, 37], it has been shown rare incidences in surrounding regions of Asia such as Korea, Taiwan, and China [38–40].

Recently, the usefulness of LRS in the diagnosis of repeat expansion disease has been gaining considerable interest in medical research [12]. Indeed, Wang et al. diagnosed patients with SCA36 using LRS [23], and it also be used for Cerebellar Ataxia, Neuropathy, and Vestibular Areflexia Syndrome (CANVAS), another repeat expansion disease [41]. It can effectively address the limitation of existing diagnostic methods such as Southern blot and NGS, which fall short in detecting large repeat expansions that cause certain SCAs. However, the challenges of complex data interpretation, coverage depth, cost, and accessibility underscore the need for further research to optimize this technology for routine clinical applications [42]. Notably, the repeat numbers of one of the expanded alleles in this study were lower than 650 repeats, the typically recognized pathogenic threshold by Southern blot [21, 43]. Despite the lower repeat number, the patient presented with characteristic clinical symptoms of SCA36 such as cerebellar ataxia, tongue fasciculation, muscle fasciculation, hearing impairment and hyperreflexia. It is uncertain whether symptoms can appear even with repeat numbers below 650, or whether the low count was due to technical differences between the Southern blot and LRS, which may have differing resolutions. Additionally, assigning repeat numbers to alleles in LRS may not be intuitive and could vary depending on algorithms, as the repeat numbers in each sequence read are distributed broadly as shown in Fig. 2, resembling the smearing pattern in Southern blot due to instability of the expansions [21, 29]. When examining the sequence structure of the repeat, it was noted that additional sequences were present between the GGCCTG repeats. However, it is unclear whether these interruptions truly exist or if they are artifacts introduced during sequencing or base calling, given the error rate of Nanopore sequencing, particularly in low-complexity regions. Future research utilizing LRS may help refine our understanding of the thresholds for repeat expansions that could trigger diseases such as SCA36. Moreover, standardization of repeat measurement will be necessary for the use of LRS as a diagnostic tool. Improving sequencing accuracy would also be beneficial for elucidating the repeat structure and its clinical significance.

This study does have certain limitations. Some participants, due to the severity of their disability, could not undergo a comprehensive examination. Furthermore, we couldn’t apply LRS to four patients suspected of having SCA36 based on RP-PCR results due to limited amounts or poor quality of DNA. Their diagnoses, however, remain strongly supported by their clinical symptoms and RP-PCR findings. Another limitation is the potential for sampling bias in cohort 1. The study cohort was derived from two hospitals in South Korea, which may restrict the generalizability of our findings to broader populations, potentially limiting the generalizability of our findings to wider populations. Furthermore, samples in cohort 2 lack thorough clinical characterization, as they were referred from various hospitals nationwide for genetic testing. This limited our access to detailed clinical information, including family history, relatedness, and comprehensive evaluations. Subsequent studies may consider incorporating a more diverse population sample to validate these findings.

The implementation of LRS with the assistance of ONT and Cas9 target enrichment emerges as a viable alternative to the traditional Southern blot method for diagnosing SCA36. This innovative approach not only demonstrates effective diagnostic capability but also circumvents some of the limitations inherent in conventional techniques. Given the considerable prevalence of SCA36 revealed in this study, incorporating this test into the routine diagnostic panel for SCAs could significantly improve the diagnostic yield, particularly in the Korean patient population. Furthermore, these findings emphasize the necessity for increased awareness and recognition of SCA36 among healthcare professionals, which could facilitate earlier diagnosis and improve patient management.

## Supplementary information

Supplemental Material

## Data Availability

The data presented in this work are available from the corresponding authors upon request.

## References

[1] Depienne C, Mandel JL. 30 years of repeat expansion disorders: What have we learned and what are the remaining challenges? Am J Hum Genet. 2021;108:764–85. 33811808 10.1016/j.ajhg.2021.03.011 PMC8205997

[2] Coutelier M, Jacoupy M, Janer A, Renaud F, Auger N, Saripella G-V, et al. NPTX1 mutations trigger endoplasmic reticulum stress and cause autosomal dominant cerebellar ataxia. Brain. 2021;145:1519–34. 10.1093/brain/awab407 34788392

[3] Paulson H. Machado-Joseph disease/spinocerebellar ataxia type 3. Handb Clin Neurol. 2012;103:437–49. 21827905 10.1016/B978-0-444-51892-7.00027-9 PMC3568768

[4] Jin DK, Oh MR, Song SM, Koh SW, Lee M, Kim GM, et al. Frequency of spinocerebellar ataxia types 1,2,3,6,7 and dentatorubral pallidoluysian atrophy mutations in Korean patients with spinocerebellar ataxia. J Neurol. 1999;246:207–10. 10323319 10.1007/s004150050335

[5] Kim JY, Park SS, Joo SI, Kim JM, Jeon BS. Molecular analysis of Spinocerebellar ataxias in Koreans: frequencies and reference ranges of SCA1, SCA2, SCA3, SCA6, and SCA7. Mol Cells. 2001;12:336–41. 11804332

[6] Lee WY, Jin DK, Oh MR, Lee JE, Song SM, Lee EA, et al. Frequency analysis and clinical characterization of spinocerebellar ataxia types 1, 2, 3, 6, and 7 in Korean patients. Arch Neurol. 2003;60:858–63. 12810491 10.1001/archneur.60.6.858

[7] Kim HJ, Jeon BS, Lee WY, Chung SJ, Yong SW, Kang JH, et al. SCA in Korea and its regional distribution: a multicenter analysis. Park Relat Disord. 2011;17:72–5. 10.1016/j.parkreldis.2010.09.006 20951073

[8] Kim JS, Kwon S, Ki CS, Youn J, Cho JW. The etiologies of chronic progressive cerebellar ataxia in a Korean population. J Clin Neurol. 2018;14:374–80. 29971977 10.3988/jcn.2018.14.3.374 PMC6032000

[9] Galatolo D, Tessa A, Filla A, Santorelli FM. Clinical application of next generation sequencing in hereditary spinocerebellar ataxia: increasing the diagnostic yield and broadening the ataxia-spasticity spectrum. A retrospective analysis. Neurogenetics. 2018;19:1–8. 29209898 10.1007/s10048-017-0532-6

[10] Rajan-Babu IS, Peng JJ, Chiu R, Study I, Study C, Li C, et al. Genome-wide sequencing as a first-tier screening test for short tandem repeat expansions. Genome Med. 2021;13:126. 34372915 10.1186/s13073-021-00932-9 PMC8351082

[11] Ibanez K, Polke J, Hagelstrom RT, Dolzhenko E, Pasko D, Thomas ERA, et al. Whole genome sequencing for the diagnosis of neurological repeat expansion disorders in the UK: a retrospective diagnostic accuracy and prospective clinical validation study. Lancet Neurol. 2022;21:234–45. 35182509 10.1016/S1474-4422(21)00462-2 PMC8850201

[12] Chintalaphani SR, Pineda SS, Deveson IW, Kumar KR. An update on the neurological short tandem repeat expansion disorders and the emergence of long-read sequencing diagnostics. Acta Neuropathol Commun. 2021;9:98. 34034831 10.1186/s40478-021-01201-x PMC8145836

[13] Kim M, Kim AR, Kim JS, Park J, Youn J, Ahn JH, et al. Clarification of undiagnosed ataxia using whole-exome sequencing with clinical implications. Park Relat Disord. 2020;80:58–64. 10.1016/j.parkreldis.2020.08.040 32961395

[14] van der Sanden B, Corominas J, de Groot M, Pennings M, Meijer RPP, Verbeek N, et al. Systematic analysis of short tandem repeats in 38,095 exomes provides an additional diagnostic yield. Genet Med. 2021;23:1569–73. 10.1038/s41436-021-01174-1 33846582

[15] Mereaux JL, Davoine CS, Coutelier M, Guillot-Noel L, Castrioto A, Charles P, et al. Fast and reliable detection of repeat expansions in spinocerebellar ataxia using exomes. J Med Genet. 2023;60:717–21. 36599645 10.1136/jmg-2022-108924

[16] Yabe I, Matsushima M, Soma H, Basri R, Sasaki H. Usefulness of the Scale for Assessment and Rating of Ataxia (SARA). J Neurol Sci. 2008;266:164–6. 17950753 10.1016/j.jns.2007.09.021

[17] Cagnoli C, Michielotto C, Matsuura T, Ashizawa T, Margolis RL, Holmes SE, et al. Detection of large pathogenic expansions in FRDA1, SCA10, and SCA12 genes using a simple fluorescent repeat-primed PCR assay. J Mol Diagn. 2004;6:96–100. 15096564 10.1016/S1525-1578(10)60496-5 PMC1867469

[18] Sato N, Amino T, Kobayashi K, Asakawa S, Ishiguro T, Tsunemi T, et al. Spinocerebellar ataxia type 31 is associated with “inserted” penta-nucleotide repeats containing (TGGAA)n. Am J Hum Genet. 2009;85:544–57. 19878914 10.1016/j.ajhg.2009.09.019 PMC2775824

[19] Ishige T, Sawai S, Itoga S, Sato K, Utsuno E, Beppu M, et al. Pentanucleotide repeat-primed PCR for genetic diagnosis of spinocerebellar ataxia type 31. J Hum Genet. 2012;57:807–8. 22992774 10.1038/jhg.2012.112

[20] Obayashi M, Stevanin G, Synofzik M, Monin ML, Duyckaerts C, Sato N, et al. Spinocerebellar ataxia type 36 exists in diverse populations and can be caused by a short hexanucleotide GGCCTG repeat expansion. J Neurol Neurosurg Psychiatry. 2015;86:986–95. 25476002 10.1136/jnnp-2014-309153

[21] Kobayashi H, Abe K, Matsuura T, Ikeda Y, Hitomi T, Akechi Y, et al. Expansion of intronic GGCCTG hexanucleotide repeat in NOP56 causes SCA36, a type of spinocerebellar ataxia accompanied by motor neuron involvement. Am J Hum Genet. 2011;89:121–30. 21683323 10.1016/j.ajhg.2011.05.015 PMC3135815

[22] Liu Q, Zhang P, Wang D, Gu W, Wang K. Interrogating the “unsequenceable” genomic trinucleotide repeat disorders by long-read sequencing. Genome Med. 2017;9:65. 28720120 10.1186/s13073-017-0456-7 PMC5514472

[23] Wang Q, Zhang C, Liu S, Liu T, Ni R, Liu X, et al. Long-read sequencing identified intronic (GGCCTG)n expansion in NOP56 in one SCA36 family and literature review. Clin Neurol Neurosurg. 2022;223:107503. 36368168 10.1016/j.clineuro.2022.107503

[24] Lee YC, Tsai PC, Guo YC, Hsiao CT, Liu GT, Liao YC, et al. Spinocerebellar ataxia type 36 in the Han Chinese. Neurol Genet. 2016;2:e68. 27123487 10.1212/NXG.0000000000000068 PMC4830187

[25] Valera JM, Diaz T, Petty LE, Quintans B, Yanez Z, Boerwinkle E, et al. Prevalence of spinocerebellar ataxia 36 in a US population. Neurol Genet. 2017;3:e174. 28761930 10.1212/NXG.0000000000000174 PMC5515602

[26] Zeng S, Zeng J, He M, Zeng X, Zhou Y, Liu Z, et al. Genetic and clinical analysis of spinocerebellar ataxia type 36 in Mainland China. Clin Genet. 2016;90:141–8. 26661328 10.1111/cge.12706

[27] Garcia-Murias M, Quintans B, Arias M, Seixas AI, Cacheiro P, Tarrio R, et al. Costa da Morte’ ataxia is spinocerebellar ataxia 36: clinical and genetic characterization. Brain. 2012;135:1423–35. 22492559 10.1093/brain/aws069 PMC3338928

[28] Lam T, Rocca C, Ibanez K, Dalmia A, Tallman S, Hadjivassiliou M, et al. Repeat expansions in NOP56 are a cause of spinocerebellar ataxia Type 36 in the British population. Brain Commun. 2023;5:fcad244. 37810464 10.1093/braincomms/fcad244 PMC10558097

[29] Lopez S, He F. Spinocerebellar Ataxia 36: from mutations toward therapies. Front Genet. 2022;13:837690. 35309140 10.3389/fgene.2022.837690 PMC8931325

[30] Teive HAG, Meira AT, Camargo CHF, Munhoz RP. The Geographic Diversity of Spinocerebellar Ataxias (SCAs) in the Americas: a systematic review. Mov Disord Clin Pr. 2019;6:531–40. 10.1002/mdc3.12822 PMC6749803 31538086

[31] Naito H, Takahashi T, Kamada M, Morino H, Yoshino H, Hattori N, et al. First report of a Japanese family with spinocerebellar ataxia type 10: The second report from Asia after a report from China. PLoS One. 2017;12:e0177955. 28542277 10.1371/journal.pone.0177955 PMC5438172

[32] Wang K, McFarland KN, Liu J, Zeng D, Landrian I, Xia G, et al. Spinocerebellar ataxia type 10 in Chinese Han. Neurol Genet. 2015;1:e26. 27066563 10.1212/NXG.0000000000000026 PMC4809459

[33] Mao C, Li X, Su Y, Luo H, Fan L, Zheng H, et al. Spinocerebellar ataxia type 10 with atypical clinical manifestation in Han Chinese. Cerebellum. 2023;22:355–62. 35441258 10.1007/s12311-022-01405-4

[34] Bahl S, Virdi K, Mittal U, Sachdeva MP, Kalla AK, Holmes SE, et al. Evidence of a common founder for SCA12 in the Indian population. Ann Hum Genet. 2005;69:528–34. 16138911 10.1046/j.1529-8817.2005.00173.x

[35] Chen Z, Wang P, Wang C, Peng Y, Hou X, Zhou X, et al. Updated frequency analysis of spinocerebellar ataxia in China. Brain. 2018;141:e22. 29444203 10.1093/brain/awy016

[36] Sakai H, Yoshida K, Shimizu Y, Morita H, Ikeda S, Matsumoto N. Analysis of an insertion mutation in a cohort of 94 patients with spinocerebellar ataxia type 31 from Nagano, Japan. Neurogenetics. 2010;11:409–15. 20424877 10.1007/s10048-010-0245-6 PMC2944954

[37] Sakakibara R, Tateno F, Kishi M, Tsuyusaki Y, Aiba Y, Terada H, et al. Genetic screening for spinocerebellar ataxia genes in a Japanese single-hospital cohort. J Mov Disord. 2017;10:116–22. 28782341 10.14802/jmd.17011 PMC5615168

[38] Lee PH, Park HY, Jeong SY, Hong JH, Kim HJ. 16q-linked autosomal dominant cerebellar ataxia in a Korean family. Eur J Neurol. 2007;14:e16–7. 17539927 10.1111/j.1468-1331.2007.01818.x

[39] Lee YC, Liu CS, Lee TY, Lo YC, Lu YC, Soong BW. SCA31 is rare in the Chinese population on Taiwan. Neurobiol Aging. 2012;33:426.e23–4. 21163552 10.1016/j.neurobiolaging.2010.10.012

[40] Ouyang Y, He Z, Li L, Qin X, Zhao Y, Yuan L. Spinocerebellar ataxia type 31 exists in northeast China. J Neurol Sci. 2012;316:164–7. 22353852 10.1016/j.jns.2012.02.005

[41] Nakamura H, Doi H, Mitsuhashi S, Miyatake S, Katoh K, Frith MC, et al. Long-read sequencing identifies the pathogenic nucleotide repeat expansion in RFC1 in a Japanese case of CANVAS. J Hum Genet. 2020;65:475–80. 32066831 10.1038/s10038-020-0733-y

[42] Mantere T, Kersten S, Hoischen A. Long-read sequencing emerging in medical genetics. Front Genet. 2019;10:426. 31134132 10.3389/fgene.2019.00426 PMC6514244

[43] Sugihara K, Maruyama H, Morino H, Miyamoto R, Ueno H, Matsumoto M, et al. The clinical characteristics of spinocerebellar ataxia 36: a study of 2121 Japanese ataxia patients. Mov Disord. 2012;27:1158–63. 22753339 10.1002/mds.25092

