# Supplementary material for: Another common genetic ataxia in South Korea: Spinocerebellar ataxia 36
Source: Eur J Hum Genet. 2025 Feb 24;34(4):515–21. doi: 10.1038/s41431-024-01783-9 (PMC13046802; doi:10.1038/s41431-024-01783-9)
Supplement: Supplementary file 1 — Supplemental Material [file 41431_2024_1783_MOESM1_ESM.docx]

Supplementary Information

**Another Common Genetic Ataxia in South Korea: Spinocerebellar ataxia 36**

Jong Hyeon Ahn, MD^1,2^, Seungbok Lee, MD, PhD^3,4^, Jangsup Moon, MD, PhD^3,5^, Yoojung Han^6,7^, Hyeshik Chang^6,7,8^ Jinyoung Youn, MD, PhD^1^, Jin Whan Cho, MD, PhD^1,2†^, Ja-Hyun Jang MD, PhD^9†^

1. Figure S1 - Distribution of repeat numbers of SCA10, SCA12, and SCA36 in Korean patients with ataxia (page 2)

2. Figure S2 - Waterfall plots illustrating the repeat structure of NOP56 alleles found in Korean patients with SCA36 (page 3)

3. Table S1 - Information for rare SCA loci (page 4)

4. Table S2 - Testing methods and primers used in the study (page 5)

5. Table S3 - Statistics of Cas9-mediated long-read sequencing (page 6)

6. Table S4 - Haplotypes of the expanded *NOP56* alleles in SCA36 patients from this study and previous reports (page 7-8)

7. Table S5 - Test items and their results in cohort 2 (page 9-10)


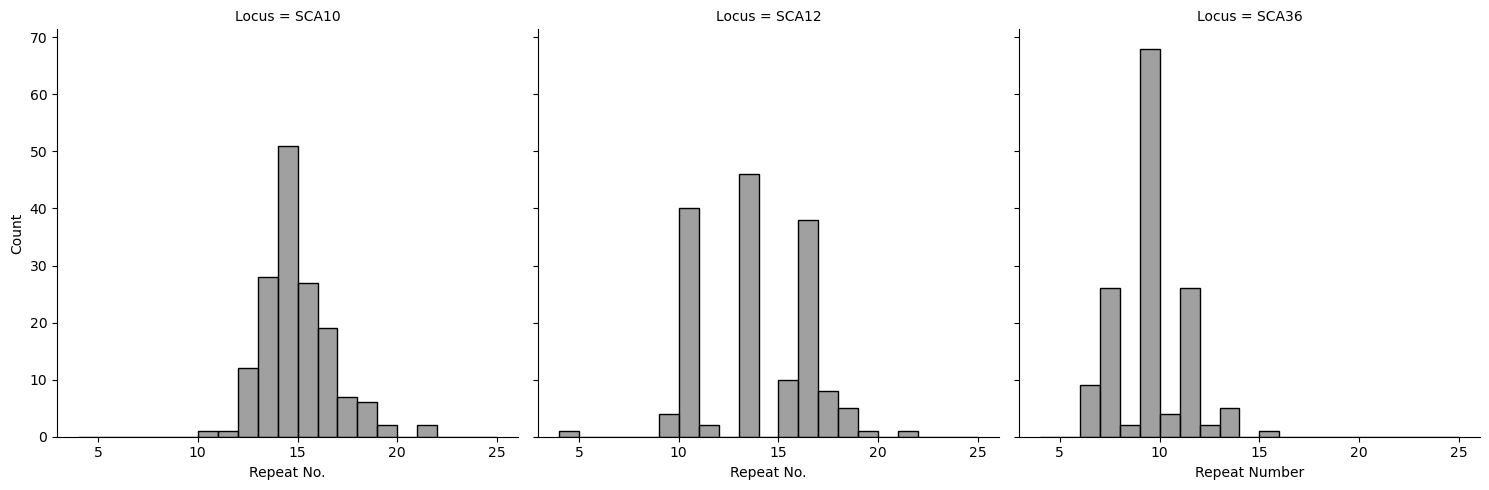


**Figure S1.** **Distribution of the repeat numbers of SCA10, SCA12, and SCA36 in Korean patients with ataxia.** The data included normal alleles found in 78 individuals from cohort 1.


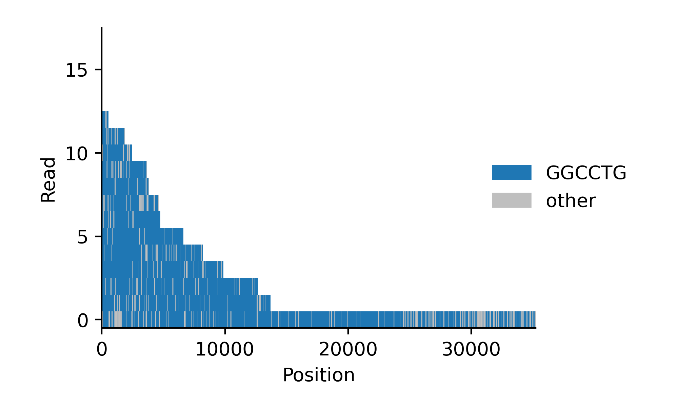

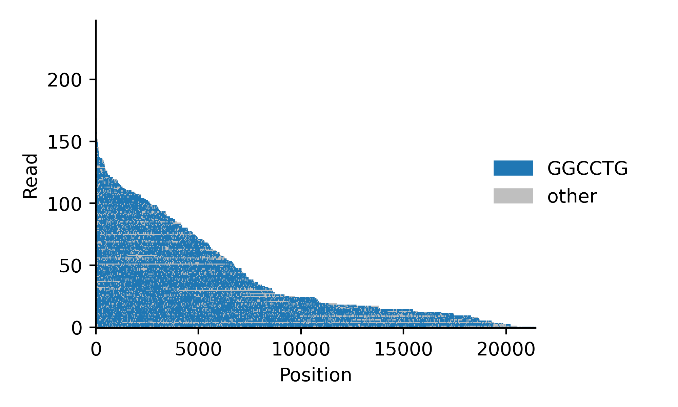


**E**

**F**

**G**

**C-1**


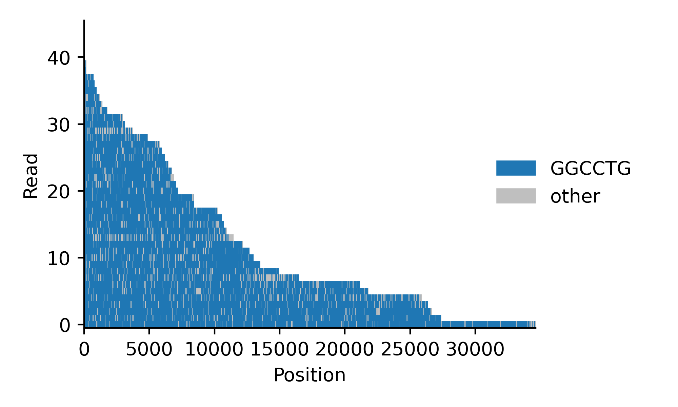

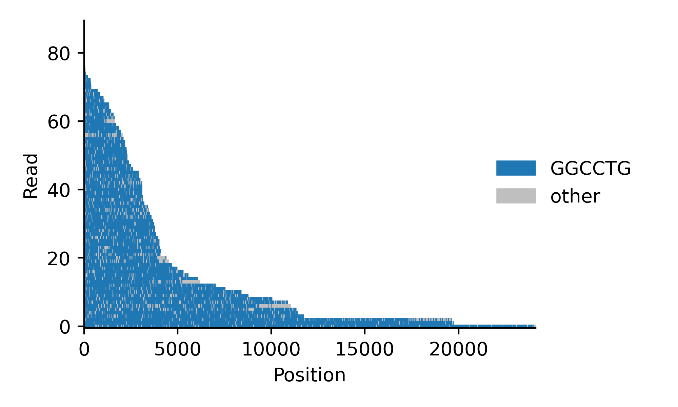


**I**

**H**


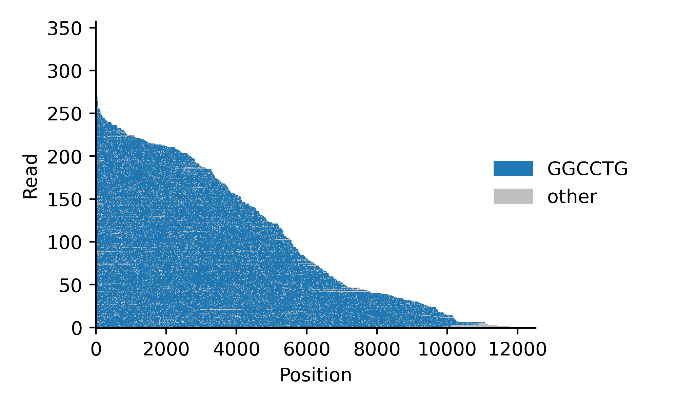

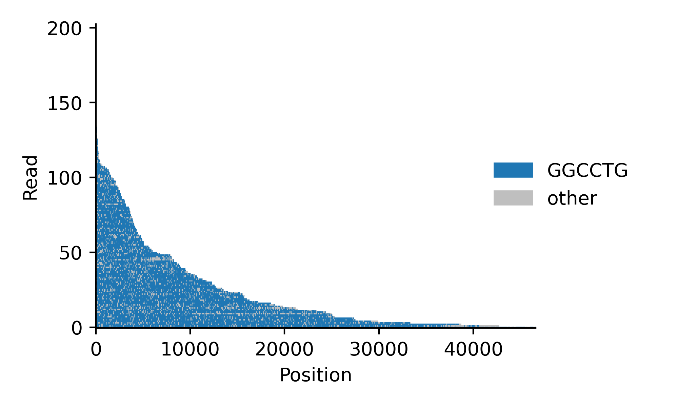


**ATA-109**


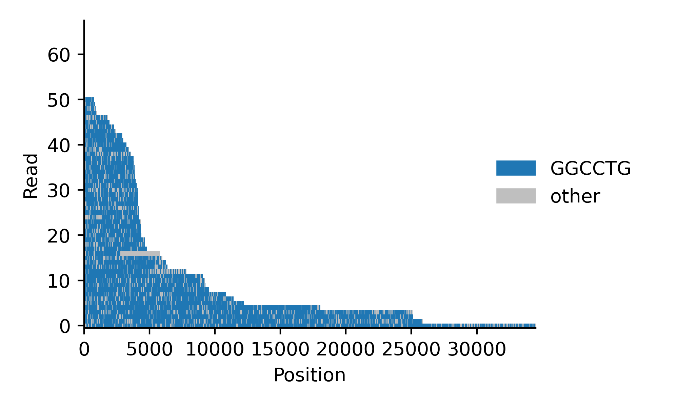


**Figure S2.** **Waterfall plots illustrating the repeat structure of NOP56 alleles found in Korean patients with SCA36.** The Y-axis represents the number of sequences, while the X-axis indicates the length of the repeat expansions in base pairs. Each plot depicts data from seven patients from cohorts 1 and 2, who underwent targeted long-read sequencing using Nanopore technology.

**Table S1. Information for rare SCA loci**

|  | **Gene** | **Location** | **Repeat motif** | **Normal** | **Full penetrance** |
| --- | --- | --- | --- | --- | --- |
| SCA10 | *ATXN10* | Intron 9 | ATTCT | 10-32 | > 800 |
| SCA12 | *PPP2R2B* | Promoter | CAG | 7-31 | 51-78 |
| SCA31 | *BEAN* | Intron 6 | TGGAA | 0 | 2.5-3.8 kb insertion |
| SCA36 | *NOP56* | Intron 1 | GGCCTG | 3-14 | > 650 |

Based on GeneReviews (https://www.ncbi.nlm.nih.gov/books/NBK1138/)

**Table S2. Testing methods and primers used in the study**

| **Loci** | **Gene** | **Testing method** | **Primers** | **Reference** |
| --- | --- | --- | --- | --- |
| SCA10 | *ATXN10* | AL-PCR | 5’-CAGATGGCAGAATGATAAACTCAA-3’ | [17] |
|  |  |  | 5’-AGCCTGGGCAACATAGAGAGA-3’ |  |
|  |  | RP-PCR | 5'-CAGATGGCAGAATGATAAACTCAA-3' |  |
|  |  |  | 5′-TACGCATCCCAGTTTGAGACG-3′ |  |
|  |  |  | 5'-TACGCATCCCAGTTTGAGACGAGAATA  GAATAGAATAGAATAGAAT-3' |  |
| SCA12 | *PPP2R2B* | AL-PCR | 5’-TGCTGGGAAAGAGTCGTG-3' | [17] |
|  |  |  | 5’-GCCAGCGCACTCACCCTC-3' |  |
|  |  | RP-PCR | 5'-TGCTGGGAAAGAGTCGTG-3' |  |
|  |  |  | 5′-TACGCATCCCAGTTTGAGACG-3′ |  |
|  |  |  | 5'-TACGCATCCCAGTTTGAGACG CTGCTGCTGCTGCTG-3’ |  |
| SCA31 | *BEAN* | Long-range PCR &  enzyme digestion | 5’-ACTCCAACTGGGATGCAGTTTCTCAAT-3' | [18] |
|  |  |  | 5’-TGGAGGAAGGAAATCAGGTCCCTAAAG-3' |  |
|  |  | RP-PCR | 5'-ACTCCAACTGGGATGCAGTTTCTCAAT-3' | [19] |
|  |  |  | 5'-TACGCATCCCAGTTTGAGACG-3' |  |
|  |  |  | 5'-TACGCATCCCAGTTTGAGACGTTC CATTCCATTCCATTCCATTCCA-3' |  |
| SCA36 | *NOP56* | AL-PCR | 5’-TTTCGGCCTGCGTTCGGG-3' | [20] |
|  |  |  | 5’-ATCTAGAGCTTTCCAGGCCC-3' |  |
|  |  | RP-PCR | 5’-TTTCGGCCTGCGTTCGGG-3' | [21] |
|  |  |  | 5’-TACGCATCCCAGTTTGAGACG-3' |  |
|  |  |  | 5’-TACGCATCCCAGTTTGAGACGCAGGCCC  AGGCCCAGGCCCAGGCC-3' |  |
|  |  | LRS | 5’- TGGCTCATTGATTGACCCTC-3’* | This study |
|  |  |  | 5’- TTACCTGACACGCATAGAGA-3’* |  |

Abbreviations: AL-PCR, allele length PCR; RP-PCR, repeat-primed PCR; LRS, long-read sequencing. * CRISPR RNA sequences used for target enrichment during the library preparation process.

**Table S3. Statistics of CRISPR/Cas9-mediated long-read sequencing by Oxford Nanopore Technology**

| **Cohort** | **Subject** | **Number of Reads (X)** | | | **Output (kb)** | | | **Mean Read Length (bp)** | | |
| --- | --- | --- | --- | --- | --- | --- | --- | --- | --- | --- |
|  |  | **On-target** | **Non-target** | **All** | **On-target** | **Non-target** | **All** | **On-target** | **Non-target** | **All** |
| 1 | C-1 | 46 | 64,108 | 64,154 | 173 | 125,402 | 125,575 | 6,521 | 1,999 | 2,003 |
|  | E | 776 | 49,358 | 50,134 | 2,792 | 273,951 | 240743 | 5,407 | 4,812 | 4,821 |
|  | F | 121 | 6,178 | 6,299 | 454 | 85,624 | 86,079 | 7,084 | 14,123 | 13,988 |
|  | G | 177 | 13,695 | 13,872 | 930 | 166,075 | 167,006 | 7,656 | 12,281 | 12,222 |
|  | H | 845 | 81,152 | 81,997 | 3,017 | 804,786 | 807,804 | 5,496 | 9,915 | 9,869 |
|  | I | 544 | 50,561 | 51,105 | 2,122 | 564,169 | 566,292 | 5,979 | 11,208 | 11,152 |
| 2 | ATA-109 | 143 | 33,930 | 34,073 | 583 | 217,894 | 218,477 | 6,886 | 6,417 | 6,419 |

The data include seven patients from cohorts 1 and 2 who underwent targeted long-read sequencing using Nanopore technology. Each library from a single patient was subjected to on a MinION flow-cell.

**Table S4. Haplotypes of the expanded *NOP56* alleles in SCA36 patients from this study and previous reports**

| **Distance  from the**  **repeats** | **Marker** | **Genomic**  **position  (GRCh38)** | **This study** | | | | | | | **Previous study** | | **Allele frequency** | | |
| --- | --- | --- | --- | --- | --- | --- | --- | --- | --- | --- | --- | --- | --- | --- |
|  |  |  | **C-1** | **E** | **F** | **G** | **H** | **I** | **ATA**  **-109** | **Japanese**  **& French**  **[20]** | **Han**  **Chinese**  **[24]** |  | **Global** | **East Asian** |
| -2,017 | rs4296499 | 2,650,741 | C | C | C | C | C | C | C | NA | NA | C= | 0.8419 | 0.8530 |
| -1,996 | rs4289262 | 2,650,762 | C | C | C | C | C | C | C | NA | NA | C= | 0.5078 | 0.3916 |
| -1,889 | rs4365539 | 2,650,869 | T | T | T | T | T | T | T | NA | NA | T= | 0.8423 | 0.8531 |
| -1,840 | rs1020640021 | 2,650,918 | T | T | T | T | T | T | T | NA | NA | T= | 0.9438 | 0.7465 |
| -1,837 | rs199813997 | 2,650,921 | T | T | T | T | T | T | T | NA | NA | T= | 0.6064 | 0.6715 |
| -1,598 | rs4815466 | 2,651,160 | G | G | G | G | G | G | G | NA | NA | G= | 0.5071 | 0.3910 |
| -1,483 | rs9680034 | 2,651,275 | G | G | G | G | G | G | G | NA | NA | G= | 0.9158 | 0.7293 |
| -1,394 | rs1883977 | 2,651,364 | T | T | T | T | T | T | T | NA | NA | T= | 0.8473 | 0.8537 |
| -1,229 | rs36159668 | 2,651,529 | C | C | C | C | C | C | C | NA | NA | C= | 0.5089 | 0.3931 |
| -1,192 | rs4813587 | 2,651,566 | T | T | T | T | T | T | T | NA | NA | T= | 0.5132 | 0.3915 |
| -1,076 | rs3827073 | 2,651,682 | C | C | C | C | C | C | C | NA | NA | C= | 0.5075 | 0.3917 |
| -731 | rs6050906 | 2,652,027 | G | G | G | G | G | G | G | NA | NA | G= | 0.8384 | 0.8526 |
| -705 | rs6050907 | 2,652,053 | A | A | A | A | A | A | A | NA | NA | A= | 0.3562 | 0.3867 |
| -428 | rs6083954 | 2,652,330 | T | T | T | T | T | T | T | T | NA | T= | 0.5374 | 0.4308 |
| -348 | rs2073196 | 2,652,410 | G | G | G | G | G | G | G | G | G | G= | 0.9134 | 0.7708 |
| -290 | rs6083956 | 2,652,468 | T | T | T | T | T | T | T | T | T | T= | 0.7988 | 0.8152 |
| -272 | rs2073195 | 2,652,486 | G | G | G | G | G | G | G | G | NA | G= | 0.7970 | 0.8212 |
| -170 | rs6115305 | 2,652,588 | G | G | G | G | G | G | G | G | NA | G= | 0.7894 | 0.8239 |
| -160 | rs4815467 | 2,652,598 | C | C | C | C | C | C | C | C | NA | C= | 0.7896 | 0.8232 |
| -108 | rs6138678 | 2,652,650 | C | C | C | C | C | C | C | NA | NA | C= | 0.1688 | 0.2233 |
| -24 | rs6115306 | 2,652,734 | G | G | G | G | G | G | G | NA | NA | G= | 0.7933 | 0.9332 |
| 0 | **REPEAT** | 2,652,758 |  |  |  |  |  |  |  |  |  |  |  |  |
| 438 | rs73070665 | 2,653,196 | T | T | T | T | T | T | T | NA | NA | T= | 0.8452 | 0.9641 |
| 532 | rs2073194 | 2,653,290 | C | C | C | C | C | C | C | NA | NA | C= | 0.8066 | 0.8246 |
| 763 | rs6050911 | 2,653,521 | C | C | C | C | C | C | C | C | C | C= | 0.7268 | 0.7389 |
| 881 | rs60145378 | 2,653,639 | I | I | I | I | I | I | I | NA | I | I= | 0.8497 | 0.9700 |
| 1,608 | rs3818060 | 2,654,366 | T | T | T | T | T | T | T | NA | NA | T= | 0.9129 | 0.7684 |
| 1,808 | rs2273137 | 2,654,566 | A | A | A | A | A | A | A | NA | NA | A= | 0.9134 | 0.7683 |
| 5,175 | rs6753 | 2,657,933 | NA | NA | NA | NA | NA | NA | NA | NA | T | T= | 0.7184 | 0.7335 |

The haplotypes of this study include seven patients from cohorts 1 and 2 who underwent targeted long-read sequencing using nanopore technology. Markers represent single nucleotide variations, except rs60145378, with a alternative allele frequency of 0.05 or higher in the gnomAD database (version 4.1.0). Indel variants were excluded due to the high error rate in nanopore sequencing; however, rs60145378 was included to allow for comparison with the previous study. Abbreviations: I, insertion; NA, not available.

**Table S5. Test items and their results in cohort 2**

| **No.** | **Subject** | **Test item** | **Result** | **No.** | **Subject** | **Test item** | **Result** |
| --- | --- | --- | --- | --- | --- | --- | --- |
| **1** | ATA-01 | SCA1, 2, 3, 6, 7, 8, 17 | ND | **51** | ATA-56 | SCA17 | ND |
| **2** | ATA-02 | SCA1, 2, 3, 6, 7, 8, 17 | ND | **52** | ATA-57 | SCA1, 2, 3, 6, 7, 8, 17 | ND |
| **3** | ATA-03 | SCA1, 2, 3, 6, 7, 8, 17 | ND | **53** | ATA-58 | SCA1, 2, 3, 6, 7, 8, 17 | ND |
| **4** | ATA-04 | SCA1, 2, 3, 6, 7, 8, 17 | ND | **54** | ATA-59 | SCA1, 2, 3, 6, 7, 8, 17 | ND |
| **5** | ATA-06 | SCA1, 2, 3, 6, 7, 8, 17 | ND | **55** | ATA-60 | SCA1, 2, 3, 6, 7, 8, 17 | ND |
| **6** | ATA-07 | SCA1, 2, 3, 6, 7, 8, 17 | ND | **56** | ATA-61 | SCA1, 2, 3, 6, 7, 8, 17 | SCA2 |
| **7** | ATA-10 | SCA1, 2, 3, 6, 7, 8, 17 | ND | **57** | ATA-62 | SCA1, 2, 3, 6, 7, 8, 17 | ND |
| **8** | ATA-12 | SCA17 | ND | **58** | ATA-63 | SCA1, 2, 3, 6, 7, 8, 17 | ND |
| **9** | ATA-13 | SCA17 | ND | **59** | ATA-64 | SCA1, 2, 3, 6, 7, 8, 17 | ND |
| **10** | ATA-14 | SCA17 | ND | **60** | ATA-65 | SCA1, 2, 3, 6, 7, 8, 17 | ND |
| **11** | ATA-15 | SCA1, 2, 3, 6, 7, 8, 17 | ND | **61** | ATA-66 | SCA1, 2, 3, 6, 7, 8, 17 | ND |
| **12** | ATA-16 | SCA1, 2, 3, 6, 7, 8, 17 | ND | **62** | ATA-67 | SCA1, 2, 3, 6, 7, 8, 17 | ND |
| **13** | ATA-17 | SCA1, 2, 3, 6, 7, 8, 17 | ND | **63** | ATA-68 | SCA1, 2, 3, 6, 7, 8, 17 | ND |
| **14** | ATA-18 | SCA1, 2, 3, 6, 7, 8, 17 | ND | **64** | ATA-70 | SCA1, 2, 3, 6, 7, 8, 17 | SCA1 |
| **15** | ATA-19 | SCA8 | ND | **65** | ATA-71 | SCA17 | ND |
| **16** | ATA-20 | SCA1, 2, 3, 6, 7, 8, 17 | ND | **66** | ATA-72 | SCA1, 2, 3, 6, 7, 8, 17 | ND |
| **17** | ATA-21 | SCA1, 2, 3, 6, 7, 8, 17 | ND | **67** | ATA-73 | SCA1, 2, 3, 6, 7, 8, 17 | ND |
| **18** | ATA-22 | SCA1, 2, 3, 6, 7, 8, 17 | ND | **68** | ATA-74 | SCA1, 2, 3, 6, 7, 8, 17 | SCA2 |
| **19** | ATA-23 | SCA17 | ND | **69** | ATA-75 | SCA1, 2, 3, 6, 7, 8, 17 | SCA2 |
| **20** | ATA-24 | SCA1, 2, 3, 6, 7, 8, 17 | ND | **70** | ATA-76 | SCA1, 2, 3, 6, 7, 8, 17 | ND |
| **21** | ATA-26 | SCA1, 2, 3, 6, 7, 8, 17 | ND | **71** | ATA-77 | SCA1, 2, 3, 6, 7, 8, 17 | ND |
| **22** | ATA-27 | SCA17 | ND | **72** | ATA-78 | SCA1, 2, 3, 6, 7, 8, 17 | ND |
| **23** | ATA-28 | SCA7 | SCA7 | **73** | ATA-79 | SCA1, 2, 3, 6, 7, 8, 17 | ND |
| **24** | ATA-29 | SCA1, 2, 3, 6, 7, 8, 17 | SCA8 | **74** | ATA-80 | SCA1, 2, 3, 6, 7, 8, 17 | ND |
| **25** | ATA-30 | SCA1, 2, 3, 6, 7, 8, 17 | ND | **75** | ATA-82 | SCA1, 2, 3, 6, 7, 8, 17 | ND |
| **26** | ATA-31 | SCA1, 2, 3, 6, 7, 8, 17 | ND | **76** | ATA-84 | SCA1, 2, 3, 6, 7, 8, 17 | ND |
| **27** | ATA-32 | SCA1, 2, 3, 6, 7, 8, 17 | ND | **77** | ATA-85 | SCA1, 2, 3, 6, 7, 8, 17 | ND |
| **28** | ATA-33 | SCA1, 2, 3, 6, 7, 8, 17 | ND | **78** | ATA-86 | SCA1, 2, 3, 6, 7, 8, 17 | ND |
| **29** | ATA-34 | SCA1, 2, 3, 6, 7, 8, 17 | ND | **79** | ATA-87 | SCA1, 2, 3, 6, 7, 8, 17 | ND |
| **30** | ATA-35 | SCA8 | ND | **80** | ATA-88 | SCA1, 2, 3, 6, 7, 8, 17 | ND |
| **31** | ATA-36 | SCA1, 2, 3, 6, 7, 8, 17 | SCA2 | **81** | ATA-90 | SCA1, 2, 3, 6, 7, 8, 17 | ND |
| **32** | ATA-37 | SCA1, 2, 3, 6, 7, 8, 17 | SCA2 | **82** | ATA-91 | SCA1, 2, 3, 6, 7, 8, 17 | SCA2 |
| **33** | ATA-38 | SCA8 | ND | **83** | ATA-92 | SCA1, 2, 3, 6, 7, 8, 17 | ND |
| **34** | ATA-39 | SCA1, 2, 3, 6, 7, 8, 17 | ND | **84** | ATA-93 | SCA1, 2, 3, 6, 7, 8, 17 | ND |
| **35** | ATA-40 | SCA1, 2, 3, 6, 7, 8, 17 | ND | **85** | ATA-94 | SCA1, 2, 3, 6, 7, 8, 17 | SCA2 |
| **36** | ATA-41 | SCA1, 2, 3, 6, 7, 8, 17 | ND | **86** | ATA-95 | SCA1, 2, 3, 6, 7, 8, 17 | ND |
| **37** | ATA-42 | SCA1, 2, 3, 6, 7, 8, 17 | ND | **87** | ATA-96 | SCA1, 2, 3, 6, 7, 8, 17 | ND |
| **38** | ATA-43 | SCA1, 2, 3, 6, 7, 8, 17 | ND | **88** | ATA-97 | SCA1, 2, 3, 6, 7, 8, 17 | SCA8 |
| **39** | ATA-44 | SCA17 | ND | **89** | ATA-100 | SCA1, 2, 3, 6, 7, 8, 17 | ND |
| **40** | ATA-45 | SCA1, 2, 3, 6, 7, 8, 17 | ND | **90** | ATA-101 | SCA1, 2, 3, 6, 7, 8, 17 | ND |
| **41** | ATA-46 | SCA1, 2, 3, 6, 7, 8, 17 | ND | **91** | ATA-102 | SCA1, 2, 3, 6, 7, 8, 17 | ND |
| **42** | ATA-47 | SCA1, 2, 3, 6, 7, 8, 17 | ND | **92** | ATA-103 | SCA1, 2, 3, 6, 7, 8, 17 | ND |
| **43** | ATA-48 | SCA1, 2, 3, 6, 7, 8, 17 | ND | **93** | ATA-104 | SCA1, 2, 3, 6, 7, 8, 17 | ND |
| **44** | ATA-49 | SCA1, 2, 3, 6, 7, 8, 17 | ND | **94** | ATA-105 | SCA1, 2, 3, 6, 7, 8, 17 | ND |
| **45** | ATA-50 | SCA1, 2, 3, 6, 7, 8, 17 | ND | **95** | ATA-106 | SCA1, 2, 3, 6, 7, 8, 17 | ND |
| **46** | ATA-51 | SCA1, 2, 3, 6, 7, 8, 17 | ND | **96** | ATA-108 | SCA17 | ND |
| **47** | ATA-52 | SCA1, 2, 3, 6, 7, 8, 17 | ND | **97** | ATA-109 | SCA17 | ND |
| **48** | ATA-53 | SCA1, 2, 3, 6, 7, 8, 17 | ND | **98** | ATA-110 | SCA1, 2, 3, 6, 7, 8, 17 | ND |
| **49** | ATA-54 | SCA1, 2, 3, 6, 7, 8, 17 | ND | **99** | ATA-111 | SCA1, 2, 3, 6, 7, 8, 17 | SCA8 |
| **50** | ATA-55 | SCA17 | ND |  |  |  |  |

Abbreviation: ND, not detected.
